# *Piscirickettsia salmonis* Imbalances the Innate Immune Response to Succeed in a Productive Infection in a Salmonid Cell Line Model

**DOI:** 10.1371/journal.pone.0163943

**Published:** 2016-10-10

**Authors:** Claudio A. Álvarez, Fernando A. Gomez, Luis Mercado, Ramón Ramírez, Sergio H. Marshall

**Affiliations:** 1 Laboratorio de Genética e Inmunología Molecular, Instituto de Biología, Pontificia Universidad Católica de Valparaíso, Valparaíso, Chile; 2 Fraunhofer Chile Research Foundation, Center for Systems Biotechnology, Las Condes, Santiago, Chile; Institute of Infectiology, GERMANY

## Abstract

*Piscirickettsia salmonis* is a facultative intracellular bacterium that causes the disease called “salmon rickettsial syndrome”. Attempts to control this disease have been unsuccessful, because existing vaccines have not achieved the expected effectiveness and the antibiotics used fail to completely eradicate the pathogen. This is in part the product of lack of scientific information that still lacks on the mechanisms used by this bacterium to overcome infected–cell responses and survive to induce a productive infection in macrophages. For that, this work was focused in determining if *P*. *salmonis* is able to modify the expression and the imbalance of IL-12 and IL-10 using an *in vitro* model. Additionally, we also evaluated the role the antimicrobial peptide hepcidin had in the control of this pathogen in infected cells. Therefore, the expression of IL-10 and IL-12 was evaluated at earlier stages of infection in the RTS11 cell line derived from O*ncorhynchus mykiss* macrophages. Simultaneously, the hepcidin expression and location was analyzed in the macrophages infected with the pathogen. Our results suggest that IL-10 is clearly induced at early stages of infection with values peaking at 36 hours post infection. Furthermore, infective *P*. *salmonis* downregulates the expression of antimicrobial peptide hepcidin and vesicles containing this peptide were unable to merge with the infective bacteria. Our results suggest that *P*. *salmonis* is able to manipulate the behavior of host cytokines and likely might constitute a virulence mechanism that promotes intracellular bacterial replication in leukocytes cells lines of trout and salmon. This mechanism involves the generation of an optimum environment for the microorganism and the downregulation of antimicrobial effectors like hepcidin.

## Introduction

*Piscirickettsia salmonis* is a Gram-negative bacterium and the causative agent of piscirickettsiosis, which produces chronic systemic infection and generally affects seawater-reared salmonids [1]. This pathogen is a non-motile, facultative, intracellular organism classified within the recently denominated *Piscirickettsiaceae* family of the Thriotrichales order. Although characterized as pleomorphic, *P*. *salmonis* is usually coccoid in shape and presents a diameter ranging from 0.1–1.5 μm [2,3]. This bacterium can grow in different cell-free media supplemented with either hemoglobin or Fe^+2^, and it can also infect different fish cell lines, including salmon macrophages and monocytes [4–7]. However, the mechanisms by which *P*. *salmonis* overcomes the antimicrobial activities of fish macrophages is unknown, just as is the case for other fish pathogens, including *Edwardsiella ictaluri*, *Edwardsiella tarda*, *Vibrio anguillarum*, *Yersinia ruckeri*, and *Mycobacterium marinum* [8].

The arrest of phagosome maturation or acidification processes is one of the most common mechanisms used by intracellular bacteria to replicate into phagocytes [9]. It was recently reported that *P*. *salmonis* can inhibit phagosome-lysosome fusion by secreting virulence effectors via the Dot/Icm secretion system [10,11]. Therefore, while it is clear that this mechanism is among the strategies employed by *P*. *salmonis* to survive inside infected cells, it is still completely unclear how this pathogen is able to avoid and control the immune response of the infected host.

In fish, as well as in other vertebrates, the first line of defense against pathogen invasion is the innate immune system, a system in which macrophages play a pivotal role in triggering immune responses [12]. Macrophages primarily act as antigen-presenting cells, but these cells are also responsible for most phagocytic activity, in addition to regulating the immune system cascade triggered by the secretion of proinflammatory cytokines [12]. These proinflammatory cytokines include interleukin (IL)-12, which are key components for the efficient performance of phagocytes in teleost fish, similar to the roles played by IL homologs in mammals [13,14]. In higher vertebrates, bacterial pathogens manipulate the responses of host cytokines inside macrophages as a survival strategy.

Some bacteria achieve cytokine manipulation via virulence effectors released by secretion systems. These effectors alter the balance between cytokines by increasing the expression of soluble IL-10 or by harboring this IL in the cell membrane via lipopolysaccharides [15–18]. As a result, the activation of macrophages is impaired, and IL-10 overexpression counteracts the expected responses of the innate immune system. This strategy is used by *Mycobacterium tuberculosis*, a well-studied intracellular bacterium that triggers an early response secretion of IL-10 in host macrophages, resulting in phagosome maturation arrest and the deactivation of additional microbicidal activities [19]. Related to this, host production of the prostaglandin E2 is involved in the induction of IL-10 as an effector of immune evasion mechanisms for important human pathogens such as *Salmonella typhi*, *M*. *tuberculosis*, *Legionella pneumophila*, and *Francisella tularensis* [18]. Furthermore, *L*. *pneumophila* and *F*. *tularensis* induce the expression of IL-12 by decreasing the pathogen levels of the p40 mRNA subunit [20,21]. Notwithstanding these advances, knowledge on the role that cytokines play in fish macrophages in avoiding or modulating intracellular pathogen infection is still limited.

Therefore, the aim of the present work was to determine if *P*. *salmonis* is able to modify and imbalance the expression of IL-12 and IL-10 in an *in vitro* fish model, specifically the permissive RTS11 cell line derived from rainbow trout (O*ncorhynchus mykiss*) macrophages. Additionally, the antimicrobial peptide hepcidin was evaluated to determine its activity during the pathogenic infection of macrophages. The obtained results suggest that *P*. *salmonis* is able to manipulate the behavior of host cytokines by increasing IL-10 expression in the RTS11 cell line. Concomitantly, hepcidin was downregulated in infected macrophages, in contrast with that observed in cells infected with the inactivated pathogen. Therefore, it appears that *P*. *salmonis* is able to selectively modulate host innate immune signaling through synergistic complementary alternatives that prevent cell interference in successful infection. In summary, this complex modulation process was observed to involve an imbalance between two target cytokines, IL-10 and IL-12, as well as the downregulation of the antibacterial peptide hepcidin. This is the first report to approximate the strategies employed by a fish bacterium to overwhelm the cell immune response, thus providing novel information which could be applied towards developing control alternatives for *P*. *salmonis* in the field.

## Materials and Methods

### Peptide Synthesis, Purification, and Characterization

Peptides were designed based on the available *O*. *mykiss* IL-10 sequence (GenBank Accession Number: NP_001232028.1). To define the best antigenic epitopes, the Kolaskar and Tongaonkar method was used in the bioinformatics server of the Immunomedicine Group of the Universidad Complutense de Madrid [http://imed.med.ucm.es/Tools/antigenic.pl] [22]. The ExPASy ProtScale tool was used to analyze the physicochemical behavior of the antigenic sequences and to identify regions of high hydrophilicity and mean flexibility [23]. The antigenic regions along the entire molecule were automatically modeled using a human homology model in the SWISS-MODEL server [24] (Fig A in S1 File). The best epitope peptides were synthesized by the solid phase multiple peptide system using Fmoc amino acids (0.65 meq/g Rink-Resin; Iris Biotech) [25], cleaved with trifluoroacetic acid/triisopropylsilane/ultrapure water (95/2.5/2.5; Novabiochem, Merck Millipore), and purified through reversed-phase high-performance liquid chromatography with a 0–70% acetonitrile-water mixture gradient for 30 min at a flow rate of 1 mL/min. The peptides were lyophilized and analyzed by matrix assisted laser desorption/ionization mass spectrometry to confirm molecular mass (Fig B in S1 File).

### Antibody Production and Characterization

Polyclonal antibodies were generated against the IL-10 epitope peptide in six week-old female CF-1 mice as previously reported [26,27]. Antiserum specificities were evaluated through an indirect enzyme-linked immunosorbent assay and immunoblotting by seeding each peptide (at 1, 0.5, 0.25, and 0.125 μg) onto a nitrocellulose membrane (0.45 μm; Thermo Scientific) (Fig C and D in S1 File). The membranes were blocked and washed as previously reported [28], and then incubated with the anti-IL-10 antiserum and pre-immune antiserum for 1 h. After this, the membranes were washed and incubated with anti-mouse IgG-HRP (Thermo Scientific) at a 1:7000 dilution. The membranes were revealed with the Enhanced Chemiluminescence Western Blotting Substrate (Pierce, Thermo Scientific). Evaluations of hepcidin were performed using a previously reported specific antiserum [27,28].

### *P*. *salmonis* Growth Conditions

The *P*. *salmonis* strain LF-89 (ATCC VR-1361) was routinely grown on sheep blood agar plates supplemented with 0.1% L-cysteine and 1% glucose at 23°C, as previously reported [5,10].

### *P*. *salmonis* Infection Kinetics in RTS-11 Cell Line

The RTS11 monocyte/macrophage cell line of *O*. *mykiss* (kindly donated by Dr. Niels Bols, University of Waterloo, Canada) was cultured at 20°C in the Leibovitz’s L-15 medium (Gibco) supplemented with 15% fetal bovine serum (Gibco), as previously reported [2,29,30]. Infection kinetics were assessed at the earlier stages of infection (1, 6, 12, 24, 36, 48, 60, and 72 h) since this is the time required by *P*. *salmonis* to generate a replicative vacuole [10,31] (S1 Fig). For this, a single *P*. *salmonis* colony grown in agar plates was used to inoculate 3 ml of the BM3 medium [6], incubated at 23°C and centrifuged at 100 rpm until reaching an optical density at 600 nm of 0.3 (≈12–16 h). Then, 200 μL of the *P*. *salmonis* medium was used to infect the RTS11 cell line, with one cell flask used for every kinetic time point and including biological duplicates. For RNA extraction, the cells were scraped from the flask and centrifuged at 300 x *g* for 10 min. The cellular pellet was processed for RNA purification with the TRIzol LS Reagent (Invitrogen) according to the manufacturer’s instructions. The RNA concentration was measured with a Nanodrop-1000 spectrophotometer.

For protein extraction, the cells were scraped, resuspended directly in 250 μl of lysis buffer (50 mM Tris-HCl, pH 7.6; 0.3 M NaCl; 0.5% Triton x-100; 0.5M EDTA and 0.2% protease inhibitor cocktail), and incubated for 30 min on ice. The mixture was centrifuged at 13,000 rpm for 10 min at 4°C, and the supernatant was transferred to a new tube. Total protein concentration was determined using the BCA Protein Assay Kit (Pierce, Thermo Scientific).

Negative controls of RTS11 cells infected with heat-inactivated *P*. *salmonis* were used. *Piscirickettsia salmonis* bacteria were inactivated at 50°C for 15 min, and cell viability was determined using the LIVE/DEAD BacLight Bacterial Viability Kit (Invitrogen) according to the manufacturer’s instruction. The infection assays were performed as previously described.

### SDS-PAGE and Western Blotting

For SDS-PAGE analyses, 25 μg of total protein were heated at 90°C for 10 min in a Laemmli Sample Buffer and electrophoresed on a 15% Tris-glycin polyacrylamide gel at 100 V for 2 h. After electrophoresis, the gels were transferred to a 0.45 μm polyvinylidene fluoride membrane and run at 100 V for 1 h. The membranes were then incubated in 5% bovine serum albumin in phosphate buffered saline (PBS) on a shaker for 1 h at 20°C. The membranes were incubated with anti-IL-10, anti-hepcidin immune sera or commercial monoclonal anti-β-actin antibody (Sigma-Aldrich), diluted to 1:2000, and stored overnight at 20°C. The secondary anti-IgG mouse-HRP antibody (1:7000; Pierce, Thermo Scientific) was added, and the membranes were incubated at 20°C for 60 min. The membranes were revealed with the Enhanced Chemiluminescence Western Blotting Substrate (Pierce, Thermo Scientific).

### Confocal Laser-Scanning Microscopy

Confocal immunofluorescence microscopy was used to localize hepcidin in the RTS11 cell lines infected with *P*. *salmonis*. The cells were fixed on glass slides with 4% paraformaldehyde for 10 min and permeabilized with 0.5% Triton X-100 in PBS. Then, the cells were incubated with anti-hepcidin rabbit antiserum (1:500) and the presence of *P*. *salmonis* was determined mouse-specific antiserum for 1 h at 20°C [27,28,32]. The samples were thoroughly washed with PBS and incubated with a 1:1000 dilution of the anti-rabbit IgG Alexa-555 conjugated antibody and anti-mouse IgG Alexa-643 conjugated antibody (Invitrogen) for 1 h at room temperature. For membrane staining, samples were exposed to Wheat germ agglutinin (WGA)- Alexa-488 conjugated (Invitrogen) for 5 min at room temperature and then washed five times with PBS.

The images were analyzed by the Leica Application Suite Advanced Fluorescence software.

### Quantitative Real-Time PCR of Hepcidin, Cytokine and Phagosomal Components mRNA Levels

Real-time PCR assays were carried out using the BioRad Real-Time PCR System. Specific primers were used to amplify IL-10, IL-12, hepcidin, cathepsin D, and the p65 guanylate-binding protein 1 (GBP1) [10,33–36] (S1 Table). In all cases, individual cDNAs were synthesized from 2 μg of RNA pre-treated with RNase-Free DNase RQ1, (Promega), using Oligo(dT) and the M-MLV Reverse Transcriptase Enzyme (Promega) according to the manufacturer’s instructions. The qRT-PCR was performed using a total reaction volume of 20 μl for each sample, containing 2X Brilliant III SYBR Green qRT-PCR Master Mix (Agilent), 300 nM of each primer, and 1 μl of each template (1:10 dilution, in triplicate). The real-time PCRs were carried out in the CFX96 Touch Real-Time PCR Detection System (BioRad) using the following parameters: 95°C for 3 min for initial denaturation, 95°C for 15 s, and 60°C for 35 s for 40 cycles. Negative controls without cDNA were also included for every time-point. Relative mRNA expressions in the RTS11 cell line were evaluated using the 2^-ΔΔCt^ method [37]. *Elongation factor 1α* (ELF) was used as the housekeeping gene, using the forward and reverse GIM-ELF2 primers [38] and the same PCR conditions described above.

## Results and Discussion

### *P*. *salmonis* Infection Induces IL-10 Expression in the RTS-11 Cell Line

Previous research has established that *P*. *salmonis* is able to survive in different fish cell lines, including those derived from immune cells [10,30], and that the inhibition of phagosome maturation is a possible mechanism used by this pathogen to survive and replicate into immune cells [10]. Consequently, *P*. *salmonis* might be able to modulate cellular signaling to inactivate the host antimicrobial response. The selective induction of host cytokines that inhibit protective antimicrobial functions could represent a virulence mechanism that effectively deceives the host response to create an optimal environment for invading intracellular bacteria.

*In vitro* infection models are valuable for defining the effects of individual cytokines on specific cell types. The present study was carried out in a relevant *in vitr*o model based on the monocyte/macrophage RTS-11cell line, which is derived from rainbow trout spleen. Infection assays were performed using a low multiplicity of infection to mimic fish infections under field conditions, where a low bacterial inoculum arrives at a presumably naïve site.

Th1 helper cells are the host immunity effectors against intracellular bacteria and they are triggered by IL-12, but a T-helper cell type 2 cytokine profile that includes IL-10 expression promotes pathogen proliferation [39]. These important macrophage-derived cytokines are usually expressed after the phagocytosis of microorganisms, thereby maintaining a balance in the antimicrobial process.

**Fig 1:**
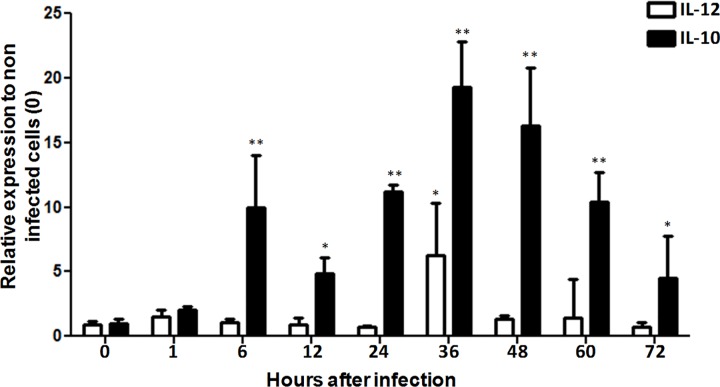
IL-12/IL-10 mRNA expression in RTS-11 cell line infected with *P*. *salmonis*. Relative mRNA expression of IL-12 and IL-10 in RTS-11 cell line infected with *P*. *salmonis* for 1, 6, 12, 24, 36, 48, 60, and 72 h. mRNA expression is expressed as a fold-change over the control (0 h). Data are presented as the mean ± standard error, with n = 4. * p < 0.05, ** p < 0.01.

**Fig 2:**
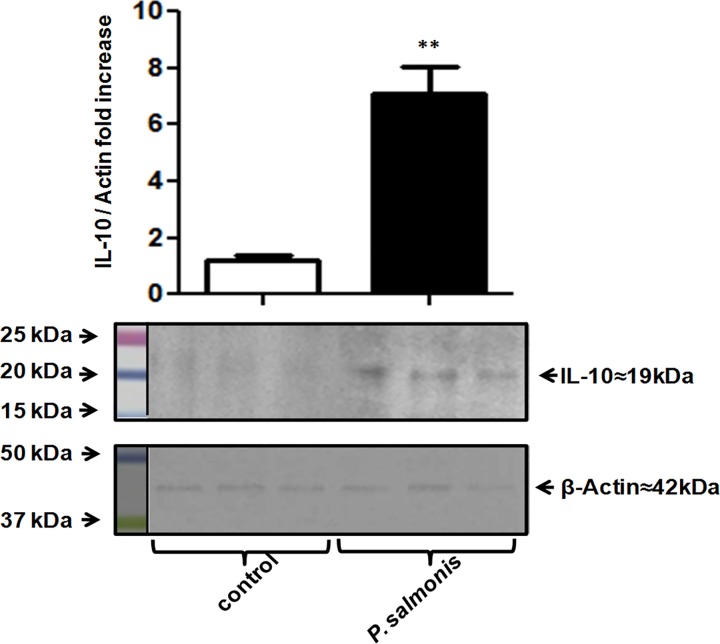
Overexpression of IL-10 during *P*. *salmonis* infection. Western blotting analysis showed upregulated IL-10 protein expression in the RTS-11 cell line 12 h after *P*. *salmonis* infection or treatment with inactivated *P*. *salmonis*. Protein bands were quantified and normalized to β-actin using the ImageJ software. Relative expression is shown as a fold-change over the uninfected cells. * p < 0.05, ** p < 0.01.

Considering the key roles of ILs in the immune response, the expressions of IL-10 and IL-12 were evaluated over the course of early *P*. *salmonis* infection of the RTS-11cell line. Results obtained through qRT-PCR showed that IL-10 mRNA was clearly induced during the early stages of infection, with values peaking at 36 h post infection (Fig 1). Consequently, IL-12 expression was not significantly altered in cells infected with virulent *P*. *salmonis*. The increase of IL-10 expression was confirmed at the protein level using a laboratory-developed anti-IL-10 antiserum (Fig 2 and S1 Fig). In contrast, IL-10 expression was undetectable at the protein level in cells infected with heat-inactivated *P*. *salmonis* (Fig 2). This result indicates that *P*. *salmonis* may manipulate the host cytokine response to upregulate IL-10, thus deactivating macrophages to permit enhanced microbial growth and, ultimately, survival within macrophages.

The inactivation of phagocytes by IL-10 induction has been previously demonstrated in other intracellular pathogens [18,40–42]. Indeed, IL-10 is a potent inhibitor of macrophage functions [43], apoptosis, cytokine synthesis, and respiratory bursts [44–46]. Additionally, the exogenous administration of IL-10 to macrophage or monocyte cultures infected with *Coxiella burnetii* or *L*. *pneumophila* promotes the replication of these intracellular bacteria [41,42]. Therefore, macrophage inactivation through the overexpression of IL-10 prevents induction of the inflammatory response, thus allowing the survival and multiplication of *P*. *salmonis*. However, the bacterial effector proteins implicated in this process remain completely unknown.

Many Gram-negative bacteria use the secretion system to deliver effectors into host cells, thereby altering the physiological functions of phagocytes [47]. The *Shigella flexneri* effector protein OspF can dephosphorylate nucleus-located mitogen-activated protein kinases that are necessary for histone H3 phosphorylation. This increases accessibility to the transcription factor NF-κB, which consequently uses the bacterial effector proteins to repress the innate immune response [48]. Another case is *Bortedella* spp., which translocates the BopN effector into the host cell via the type III secretion system, where it induces enhanced IL-10 production through the downregulation of mitogen-activated protein kinases [15]. Likewise, pathogenic *Aeromonas* and *Yersiniae* species possess a functional type III secretion system that delivers proteins into the cytoplasm of host cells [49]. Therefore, bacterial pathogens use effector proteins to enable survival against and dominance of the host innate immune.

Interestingly, *P*. *salmonis* possesses a functional Dot/Icm type IV-B secretion system as well as associated effector proteins [10]. Additionally, phagosome acidification induces the intracellular overexpression of the *P*. *salmonis dot/icm* genes. Considering this, it is possible that phagosome acidification triggers the events that secrete protein effectors via the Dot/Icm system, ultimately favoring intracellular bacterial replication [10]. Furthermore, a Dot/Icm effector of *P*. *salmonis* is likely secreted before lysosome-phagosome fusion takes place and, consequently, could participate in regulating the cellular signals of bacteria-infected phagocytes. This would finally prevent pathogen destruction by phagocytes, but further studies are needed to confirm this hypothesis.

### *P*. *salmonis* Infection Modulates the Expression of Phagosomal Components

The initial morphological event in the host-bacteria interaction is the internalization of the bacterial pathogen. This event involves GTPases that participate in moving pathogen-containing vesicles (phagosomes) and in relocating antimicrobial molecules so that the pathogen can exert its actions [50,51]. To determine whether *P*. *salmonis* could alter the expression of phagosomal components during the early stages of infection, the mRNA expressions of GBP1 GTPase, cathepsin D protease, and hepcidin, an antimicrobial peptide, were determined in the RTS-11 cell line.

**Fig 3:**
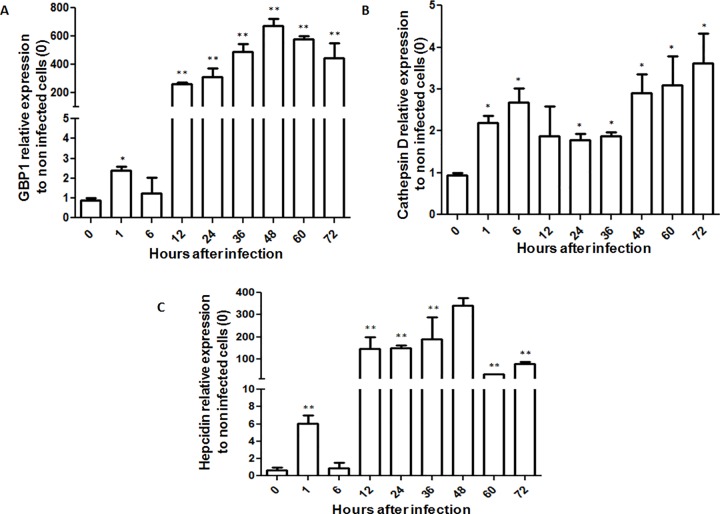
mRNA expression of phagosomal components in RTS-11 cell line infected with *P*. *salmonis*. Relative mRNA expression of **(A)** GBP1, **(B)** Cathepsin D, and **(C)** hepcidin in RTS-11 cell line infected with *P*. *salmonis* for 1, 6, 12, 24, 36, 48, 60, and 72 h. mRNA expression is expressed as a fold-change over the control (0 h). Data are presented as the mean ± standard error, with n = 4. * p < 0.05, ** p < 0.01.

The results of qRT-PCR analysis showed increased GBP1 expression 12 h post infection (Fig 3A). These results suggest that GBP1 mRNA expression is regulated by *P*. *salmonis* after bacterial internalization. In *L*. *pneumophila*, GBPs are necessary for the recognition of cytoplasmic lipopolysaccharides derived from this intracellular bacterial pathogen [52]. Additionally, GBP1 participates in the vesicle trafficking processes required for phagosomal movement, probably by inducing actin remodeling via globular actin sequestering and/or filament capping [53]. Related to this, it was recently found that *P*. *salmonis* can exploit actin monomers to disorganize the cytoskeleton and to *de novo* synthesize actin [31]. Therefore, GBP1 is probably involved in the macrophage cytoskeleton disruption promoted by *P*. *salmonis*.

On the other hand, cathepsin D mRNA was increase at minor level by *P*. *salmonis* (Fig 3B). This protease is needed for the destruction of microorganisms in the phagolysosome. However, *P*. *salmonis* is able to inhibit phagosome-lysosome fusion, possibly through the targeted expression and effector secretion of the Dot/Icm system and/or through another virulence mechanism, thereby ensuring pathogenic multiplication in the infected cells [10]. Additionally, IL-10 decreases the expression of cathepsin D in monocytes, resulting in the decreased delivery of cathepsin D to lysosomes [54].

### *P*. *salmonis* Inhibits the Actions of Hepcidin in Macrophages

Antimicrobial peptides are an important component of the innate immune system and are necessary for eliminating pathogenic microorganisms. A previous report found that hepcidin, an antimicrobial peptide, is able to decrease *P*. *salmonis* growth [26]. Moreover, a low concentration of synthetic trout hepcidin can inhibit *P*. *salmonis* replication in a liquid medium by occupying a mechanism independent from bacteria-induced membrane disruption, probably by binding to an intracellular target [26].

Hepcidin is expressed in macrophages or cells with phagocytic capacity, and hepcidin expression increases after viral, fungal, or bacterial infection [55,56]. Particularly, infection of the RTS-11 cell line by the infectious hematopoietic necrosis virus results in increased hepcidin mRNA expression in the first hours post infection, but, interestingly, expression decreases over the course of infection [33]. In the present study, hepcidin mRNA was upregulated 12 h post infection with *P*. *salmonis* (Fig 3C). Therefore, hepcidin could be an important host defense effector against viral and bacterial infection in salmonids. To more clearly elucidate the role of hepcidin in the immune response of a salmonid species, the effect of *P*. *salmonis* infection on hepcidin expression in the RTS-11 cell line was compared against a control containing heat-inactivated *P*. *salmonis*.

**Fig 4:**
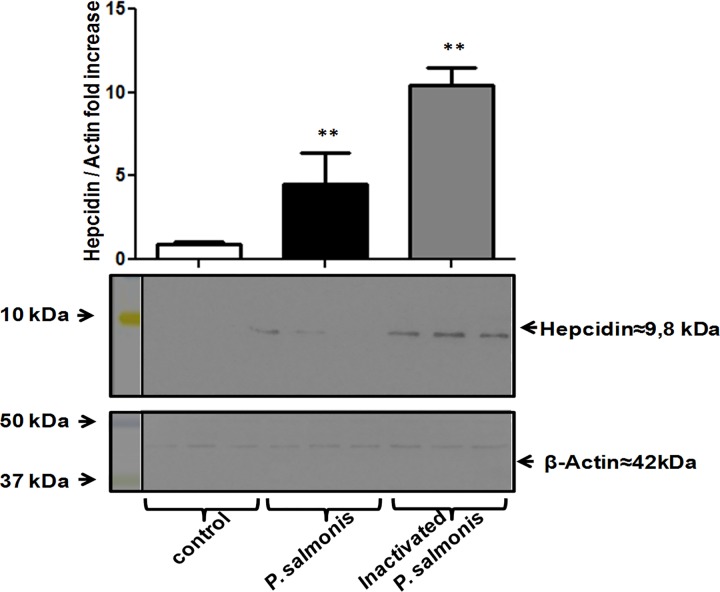
Effect of *P*. *salmonis* infection on hepcidin protein expression in RTS-11 cells. Western blotting analysis evidenced a change in hepcidin protein expression in the RTS-11 cell line 12 h after *P*. *salmonis* infection or treatment with inactivated *P*. *salmonis*. Protein bands were quantified and normalized to β-actin using the ImageJ software. Relative expression is shown as a fold-change over uninfected cells. * p < 0.05, ** p < 0.01.

A specific antiserum was used to assess the expression of hepcidin at the protein level [27,28], and relative expression was measured using actin as a housekeeping protein. Western blot analysis showed that *P*. *salmonis* infection increase the hepcidin expression more slowly than the RTS-11 cell line after exposure to heat-inactivated *P*. *salmonis* (Fig 4). Considering that intracellular pathogens can redirect the traffic of host vesicles using different mechanisms of host-defense evasion [57], focus was given to evaluating the localization of hepcidin in adherent cells of the RTS11 cell line 12 h post infection. The infected cells were stained with propidium iodide to detect bacterial and cell DNA. The infected cells were also stained with specific anti-*P salmonis* or anti-hepcidin antisera to evaluate possible co-localization with phagosome-containing bacteria. Samples were analyzed under a confocal laser-scanning microscope.

**Fig 5:**
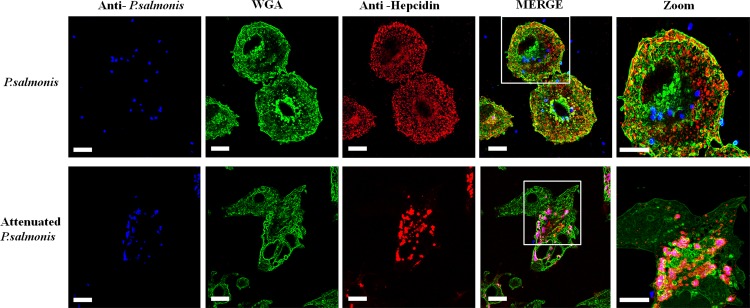
Localization of hepcidin and *P*. *salmonis* in the RTS-11 cell line. **(A)** RTS-11 cell line infected with *P*. *salmonis*. **(B)** RTS-11 cell line infected with inactivated *P*. *salmonis*. Hepcidin was detected using rabbit specific antiserum and the anti-rabbit IgG Alexa-555 conjugated antibody (Red) and *P*. *salmonis* was detected with a mouse-specific antiserum and an anti-mouse IgG Alexa-643 conjugated antibody (Blue). Wheat germ agglutinin (WGA)- Alexa-488 conjugated was used for membrane staining (Green). Scale bar, 10 μm.

The obtained confocal images revealed the co-localization of inactivated *P*. *salmonis* with hepcidin at 12 h post infection (Fig 5). Interestingly, when the RTS-11 cell lines were infected with infective *P*. *salmonis*, co-localization was not observed (Fig 5). The vesicles containing the antimicrobial peptide were unable to merge with the infective bacteria and were separately observed.

Pathogens that are able to inhibit phagosome-lysosome fusion, such as *P*. *salmonis*, use different mechanisms to disrupt the traffic of host vesicles through cytoskeleton destabilization [58]. Many pathogenic bacteria have the ability to reorganize the actin cytoskeleton underlying the plasma membrane, which comes into contact with the pathogen [58]. Actin cytoskeleton rearrangement allows bacteria to persist in the macrophage cytosol. A recent study demonstrated that *P*. *salmonis* can disorganize the actin cytoskeleton of the SHK-1, RTS-11, and ASK cell lines, likely as a way to favor protected bacterial replication [31]. As a result of actin cytoskeleton disorganization, phagosome-containing bacteria are inaccessible by antimicrobial peptides since host vesicle traffic is destroyed.

However, this is not the only mechanism occupied by intracellular bacteria to evade the innate effectors present in phagocytic cells. For example, *C*. *burnetii* delivers different effector proteins directly into the host cell cytoplasm through a type IV B secretion system. These effector proteins generate outer membrane vesicles that act as decoys for sequestering by antimicrobial peptides [59]. In relation to *P*. *salmonis*, recent research demonstrated that the functional Dot/Icm secretion system in *P*. *salmonis* is similar to systems in closely related pathogens, including *L*. *pneumophila* and *C*. *burnetii* [10]. Nevertheless, future studies are needed to determine if outer membrane vesicle-forming effector proteins are present in *P*. *salmonis*.

## Conclusions

Due to a lack of knowledge regarding the host innate immune system pathways modulated by *P*. *salmonis*, there has been little success in producing efficient vaccines and novel treatments against salmon rickettsial syndrome. Therefore, gaining insight into the signaling pathways and associated components that are mediated by *P*. *salmonis* during infection will greatly facilitate the development of new therapies and vaccines. In the present study, the observed upregulation of IL-10 might constitute a virulence mechanism used by *P*. *salmonis* to promote intracellular bacterial replication in salmonid macrophage cells lines. This mechanism optimized the cellular environment for the pathogenic microorganism and downregulated antimicrobial effectors, particularly hepcidin. The aims of future studies will be to replicate the currently presented assays *in vivo* using primary macrophage cultures and to analyze *in vivo* the effect of exogenous IL-10 on *P*. *salmonis* growth.

## Supporting Information

S1 Fig Growth of *P*. *salmonis* on RTS-11 line cell. qRT-PCR expression analysis of *P*. *salmonis* ITS on RTS11 cell line after 24, 48 and 72 hours post infection. Data are presented as the mean ± standard error, with n = 4.
(PDF)

S1 File Antigenic peptide and antisera production against trout IL-10. **(A)** The chosen epitope peptide (KKEIVQCRNYFSCKKPFDI) from rainbow trout IL-10. **(B)** The peptide was synthesized by a solid phase multiple peptide system, and the correct mass was obtained by matrix assisted laser desorption/ionization mass spectrometry (Theoretical Molecular Weight: 2346.81 kDa). **(C)** Dot-blot analysis of peptide serum immunized in mice. **(D)** Indirect enzyme-linked immunosorbent assay using the synthetic peptide and peptide serum.
(PDF)

S1 Table Primer sequences used in this study. (PDF)
