# Supplementary material for: Piscirickettsia salmonis Imbalances the Innate Immune Response to Succeed in a Productive Infection in a Salmonid Cell Line Model
Source: PLoS One. 2016 Oct 10;11(10):e0163943. doi: 10.1371/journal.pone.0163943 (PMC5056700; doi:10.1371/journal.pone.0163943)
Supplement: S1 Fig — qRT-PCR expression analysis of P. salmonis ITS on RTS11 cell line after 24, 48 and 72 hours post infection. Data are presented as the mean ± standard error, with n = 4. (PDF) [file pone.0163943.s001.pdf]

S1 Figure

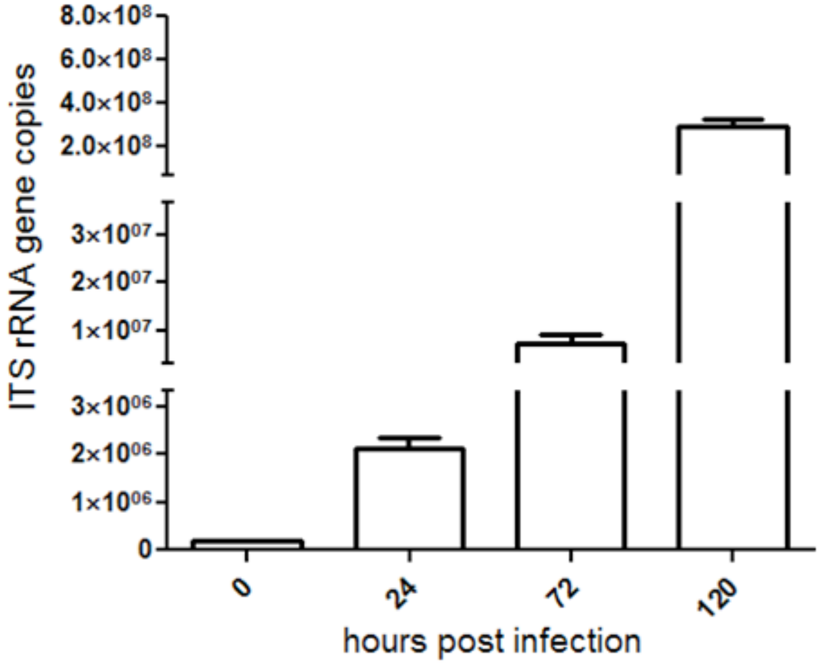

S1 Fig. Quantification of *P. salmonis* in RTS-11 line cell.

In order to corroborate a productive infection in RTS11 cells, was made an infection to determine the increase of bacterial number in time. For this purpose 6 well plates of RTS11 cells (6 million of cell per well) were infected with *P. salmonis* (inoculum of 2 x 10^4 bacteria/ml), making an infection kinetic of 24, 72 and 120 hours (all in duplicated). Bacterial multiplication was measured by qPCR using absolute quantification, using the ITS (16S-23S intergenic spacer) as has been describer previously (Olivares and Marshall, 2010). These results are shown that the bacterial number increases 1 log since the initial inoculum.
