# Supplementary figures and images for: Piscirickettsia salmonis Imbalances the Innate Immune Response to Succeed in a Productive Infection in a Salmonid Cell Line Model
Source: PLoS One. 2016 Oct 10;11(10):e0163943. doi: 10.1371/journal.pone.0163943 (PMC5056700; doi:10.1371/journal.pone.0163943)

## **S1 File**

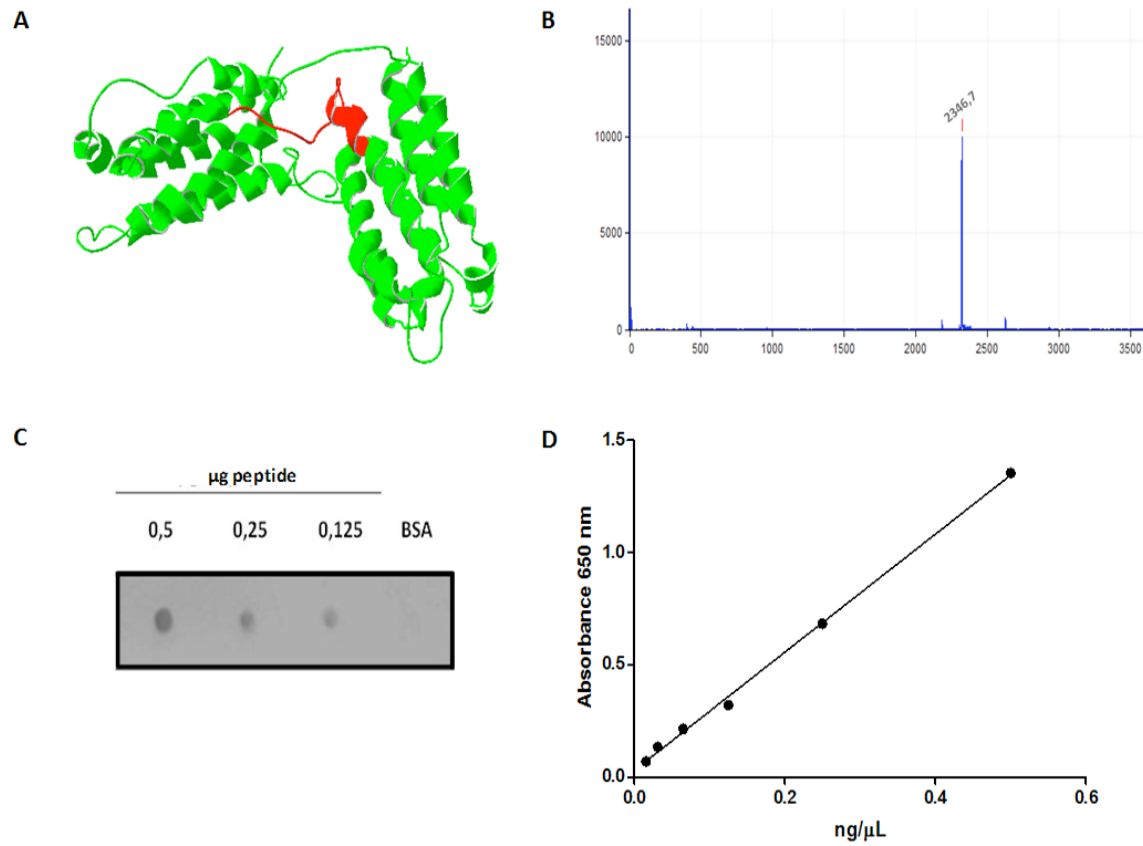

**S1 File: Antigenic peptide and antisera production against trout IL-10.**

Supplement: S1 File — (A) The chosen epitope peptide (KKEIVQCRNYFSCKKPFDI) from rainbow trout IL-10. (B) The peptide was synthesized by a solid phase multiple peptide system, and the correct mass was obtained by matrix assisted laser desorption/ionization mass spectrometry (Theoretical Molecular Weight: 2346.81 kDa). (C) Dot-blot analysis of peptide serum immunized in mice. (D) Indirect enzyme-linked immunosorbent assay using the synthetic peptide and peptide serum. (PDF) [file pone.0163943.s002.pdf]
