# Supplementary material for: Piscirickettsia salmonis Imbalances the Innate Immune Response to Succeed in a Productive Infection in a Salmonid Cell Line Model
Source: PLoS One. 2016 Oct 10;11(10):e0163943. doi: 10.1371/journal.pone.0163943 (PMC5056700; doi:10.1371/journal.pone.0163943)
Supplement: S1 Table — (PDF) [file pone.0163943.s003.pdf]

S1 Table. Primer sequences used in this study

Molecule	Primer	Sequence	Ref.
Interleukin 12	IL-12Fw	CCCAACACGGACAGGAACAC	[35]
	IL-12Rev	GCCCTTCAGTAACTTACACAGATGGAC	
Interleukin 10	IL-10Fw	GCCCTTCAGTAACTTACACAGATGGAC	[36]
	IL-10Rev	GTCGTTGTTGTTCTGTGTTCTGTTGT	
Cathepsin D	CatDFw	GCCTGTCATCACATTCAACCT	[34]
	CatDRev	CCACTCAGGCAGATGGTCTTA	
Hepcidin	HepFw	CCACTCAGGCAGATGGTCTTA	[33]
	HepRev	TTATTGCAGGCAGGTTCT	
Guanylate-binding protein 1	GBP1Fw	CGTCAATCAGCTGTCAGAGAACCA	This study
	GBP1Rev	TCGGAGGCATCCTTGTTCTGTTTG	
elongation factor 1 alpha	Elf1aFw	GTCTACAAAATCGGCGGTAT	[38]
	Elf1aRev	CTTGACGGACACGTTCTTGA	
